# Takayasu’s arteritis associated with tuberculosis: a retrospective study

**DOI:** 10.1186/s42358-024-00424-5

**Published:** 2024-11-11

**Authors:** Ana Luisa Souza Pedreira, Maria de Lourdes Castro de Oliveira Figueiroa, Mariana Oliveira Miranda, Alisson Regis de Santana, Victor Pereira Mattos, Adriane Sousa da Paz, Camila Cendon Duran, Mittermayer Barreto Santiago

**Affiliations:** 1 Hospital Universitário Professor Edgard Santos – Universidade Federal da Bahia, Salvador, Bahia Brazil; 2 Escola Bahiana de Medicina e Saúde Pública, Av. Dom João VI, 275 - Brotas, Salvador, BA 40290-000 Brazil

**Keywords:** Large-vessel vasculitis, Latent tuberculosis, Tuberculosis, Takayasu arteritis, Aortitis

## Abstract

**Background:**

Takayasu arteritis (TA) and tuberculosis (TB) share similar histopathological and immunological characteristics. Studies comparing patients with TA with or without active or latent TB infection (LTBI) have revealed some differences in clinical and angiographic profiles. Patient with TA and history of TB exhibited more constitutional symptoms and structural damage to the aorta. This study compared the clinical and radiological features of patients with TA with and without active TB or LTBI.

**Methods:**

We retrospectively analyzed the data of patients with TA at a public tertiary referral outpatient clinic in northeast Brazil from January 2017 to June 2022. Comparisons of clinical features were made according to the presence of TB.

**Results:**

Fifty patients met the eligibility criteria, and a association with TB was identified in 20 (40%) patients (active TB in six and LTBI in 14). There was a predominance of females, and the average age of patients was 40 years. Weight loss was more common in patients with TA and TB (*p* = 0.005). No significant intergroup differences were noted in terms of comorbidities, medications, erythrocyte sedimentation rates, or C-reactive protein levels. Significant differences were found in abdominal aortic involvement (25% of patients with TA and TB vs. 11.4% in subjects with TA without TB; *p* = 0.013). Dilations and aneurysms were significantly more common in patients with TA and TB (*p* = 0.009 and *p* = 0.027, respectively).

**Conclusion:**

Patients with TA and TB have a higher prevalence of dilatation and aneurysms, most commonly in the abdominal aorta.

## Background

The possible relationship between infection with *Mycobacterium tuberculosis* (MT) and Takayasu’s arteritis (TA), a primary large-vessel vasculitis, dates back to the 1940s, when Shimizu and Sano first described this condition [1].

Tuberculosis (TB) and TA share the histopathological characteristics of granulomatous lesions, and immunological studies have suggested that a molecular mimicry mechanism involving a group of proteins termed heat shock proteins underlies both [2, 3]. In addition to the fact that in numerous cases, active TB precedes the diagnosis of TA, latent TB infection (LTBI) is more prevalent in patients with TA than in individuals without other autoimmune diseases or in healthy controls [4].

In the last decade, retrospective studies have compared the clinical and angiographic characteristics of patients with TA with or without a prior history of TB. Despite the specificity of the study populations, these studies suggested differences between the groups, with patients with TA and a history of TB presenting with stronger constitutional symptoms and worse aortic structural damage [5–7].

These patients are currently being treated with immunobiological drugs, particularly those containing anti-tumor necrosis factors. Therefore, it is necessary to determine whether this treatment is directly associated with LTBI reactivation.

This study aimed to test the hypothesis that patients with TA and TB present with different clinical and radiological features from those with TA alone.

## Methods

This retrospective case-control study analyzed secondary data from the clinical, laboratory, and imaging records of patients with TA who were followed up at a public tertiary referral outpatient rheumatology center in Salvador, Bahia, Brazil, from January 2017 to June 2022. The inclusion criteria were age > 18 years and a diagnosis of TA based on the 1990 American College of Rheumatology (ACR) criteria [8]. The exclusion criteria were the presence of other concomitant autoimmune diseases and insufficient data in medical records. For the analysis, patients were divided into those with TA but without a history of active TB or LTBI and those with TA and a previous, concomitant, or subsequent diagnosis of TB. LTBI was diagnosed using a positive tuberculin skin test (> 5 mm) or interferon gamma release assay (IGRA)-assay-QuantiFERON TB test, and active TB was diagnosed using bacilloscopy, molecular rapid test for TB, or histopathology. Data extracted from medical records included signs and symptoms, laboratory test results (erythrocyte sedimentation rate [ESR] and C-reactive protein [CRP]), angiography, computed tomography or magnetic resonance angiography findings, comorbidities, and medications used. Patterns of angiographic lesions were categorized into four types: stenosis, occlusion, dilatation, and aneurysms. Stenosis was a narrowing of the vessels compared to the normal upper or lower portions. Occlusion was defined as the case where the contrast material did not pass through the vessel in the affected segment. An aneurysm was defined as a dilated artery that was more than 50% of the normally expected arterial diameter compared to upper or lower normal sites from the lesion. If a dilated lesion did not meet the criteria of an aneurysm, it was defined as dilatation [9].

Weight loss was defined as at least 10% reduction in body weight over 180 days.

Statistical analyses were performed using Statistical Package for the Social Sciences version 21 (SPSS, Chicago, IL, USA). Chi-square or Fisher’s exact tests were used to evaluate the associations between variables among the groups. The continuous variables were analyzed using the Student’s *t*-test or the Mann-Whitney U test according to their distribution.

Differences were considered statistically significant at *p* < 0.05. This study was approved by the Human Research Ethics Committee (*Comitê de Ética em Pesquisa com Seres Humanos*—CEPSH) of Bahiana School of Medicine and Public Health (*Escola Bahiana de Medicina e Saúde Pública*—EBMSP; Certificate of Presentation for Ethical Consideration [*Certificado de Apresentação de Apreciação Ética*—CAAE]: 73383717.3.0000.5544).

## Results

A total of 62 medical records were analyzed in this study, of which 50 met the eligibility criteria. Among them, an association with TB was identified in 20 patients (40% of the sample), including six patients with active TB (four pulmonary, one ganglionic, and one cutaneous) and 14 patients with LTBI (those with positive purified protein derivative skin test and/or IGRA results). Of the six patients with active TB, four were diagnosed with TB before TA, and two were concomitantly diagnosed with TB and TA. The second group included 30 patients (60%) with TA that was not associated with TB.

The groups had homogeneous clinical and epidemiological characteristics (Table 1) except for weight loss, which was more common in the TA with TB group (*p* = 0.005). No significant intergroup differences were found in comorbidities, medications used, ESR, or serum CRP levels (Tables 2, 3 and 4).

**Table 1:** Clinical and epidemiological characteristics of the patients with Takayasu arteritis with and without tuberculosis

Variables	Tuberculosis		*P* value
	Absent	Present	
**Age (years)**	43 ± 14	40 ± 12	0.438
**Age at diagnosis (years)**	33 ± 14	27 ± 8	0.056
**Sex**			
Female	7 (54.0)	17 (34.0)	0.672
**Numano classification**			
Type I	10 (20.4)	3 (6.1)	0.323
Type IIa	3 (6.1)	1 (2.0)	
Type IIb	1 (2.0)	1 (2.0)	
Type III	0 (0.0)	1 (2.0)	
Type IV	3 (6.1)	1 (2.0)	
Type V	12 (24.5)	13 (26.5)	
**Fever**	2 (4.0)	4 (8.0)	0.202
**Fatigue**	16 (32.0)	14 (28.0)	0.377
**Ponderal waste**	1 (2.0)	7 (14.0)	0.005
**Arthralgia**	18 (36.0)	8 (16.0)	0.248
**Arthritis**	2 (4.0)	0 (0.0)	0.510
**Myalgia**	12 (24.5)	8 (16.3)	1.000
**Cutaneous vasculitis**	0 (0.0)	2 (4.0)	0.155
**Erythema nodosum**	2 (4.0)	2 (4.0)	1.000
**Angina**	4 (8.0)	5 (10.0)	0.454
**Upper limbs claudication**	18 (36.0)	11 (22.0)	0.776
**Lower limbs claudication**	6 (12.0)	7 (14.0)	0.327
**Carotidynia**	9 (18.0)	3 (6.0)	0.317
**Vertigo**	12 (24.0)	8 (16.0)	1.000
**Visual disturbance**	5 (10.0)	6 (12.0)	0.311
**Amaurosis**	1 (2.0)	0 (0.0)	1.000
**Syncope**	5 (10.0)	0 (0.0)	0.075

**Table 2:** Comorbidities of patients with Takayasu arteritis with and without tuberculosis

Variables	Tuberculosis		*P* value
	No	Yes	
	*n* (%)	*n* (%)	
**Arterial hypertension**	24 (48.0)	15 (30.0)	0.736
**Renovascular hypertension**	10 (20.0)	8 (16.0)	0.765
**Cardiac failure**	3 (6.0)	4 (8.0)	0.416
**Chronic coronary syndrome**	4 (8.0)	3 (6.0)	1.000
**Acute myocardial infarction**	0 (0.0)	2 (4.0)	0.155
**Stroke**	7 (14.0)	3 (6.0)	0.720
**Peripheral artery disease**	2 (4.0)	1 (2.0)	1.000
**Chronic renal failure**	3 (6.0)	1 (2.0)	0.641
**Diabetes mellitus**	3 (6.0)	1 (2.0)	0.641

**Table 3:** Medications used in patients with Takayasu arteritis with and without tuberculosis

Variables	Tuberculosis		*P* value
	No	Yes	
	*n* (%)	*n* (%)	
**ASA**	23 (46.0)	13 (26.0)	0.522
**AH** ^**a**^			
1	2 (4.0)	5 (10.0)	0.336
2	14 (28.0)	5 (10.0)	
3	6 (12.0)	4 (8.0)	
>3	2 (4.0)	2 (4.0)	
**Statins**	8 (16.0)	9 (18.0)	0.229
**MTX** ^**a**^			
10–15 mg	6 (12.0)	1 (2.0)	0.246
16–19 mg	7 (14.0)	7 (14.0)	
20–25 mg	6 (12.0)	2 (4.0)	
**MFM**	0 (0.0)	1 (2.0)	0.400
**AZA** ^**a**^			
50 mg	0 (0.0)	1 (2.0)	0.189
100 mg	1 (2.0)	2 (4.0)	
150 mg	2 (4.0)	0 (0.0)	
**IFX**	0 (0.0)	2 (4.0)	0.155
**Prednisone** ^**a**^			
<10 mg	5 (10.0)	4 (8.0)	0.275
10–20 mg	8 (16.0)	2 (4.0)	
>20 mg	0 (0.0)	1 (2.0)	

^a^Likelihood ratio chi-square test

*ASA* acetyl salicylic acid, *AH* antihypertensive drugs, *MTX* methotrexate, *MFM* mycophenolate mofetil, *AZA* azathioprine, *IFX* infliximab

**Table 4:** Acute phase reactants in patients with Takayasu arteritis with and without tuberculosis

Variables	Tuberculosis		*P* value
	No	Yes	
**ESR** ^**a**^	27.54±19.07	30.72±17.07	0.573
**CRP** ^**b**^	2.20 (0.00–7.9)	3.4 (1.3–6.3)	0.367

^a^T test

^b^U Mann–Whitney test

*ESR* erythrocyte sedimentation rate, *CRP* C-reactive protein

Major intergroup differences were noted in the angiographic findings. Table 5 outlines these findings and reveals that abdominal aortic involvement occurred in 25% of the patients with TA and TB and in 11.4% of the patients with TA without TB (*p* = 0.013).

**Table 5:** Anatomical location of the angiography findings in the patients with Takayasu arteritis with/without tuberculosis

Variables	Tuberculosis		*P* value
	Yes	No	
	*n* (%)	*n* (%)	
**Subclavian** ^**a**^			
Left	7 (15.9%)	8 (18.2%)	0.665
Right	1 (2.3%)	4 (9.1%)	
Both	7 (15.9%)	7 (15.9%)	
**Common carotid** ^**a**^			
Left	5 (11.4%)	4 (9.1%)	0.384
Right	1 (2.3%)	3 (6.8%)	
Both	4 (9.1%)	10 (22.7%)	
**Internal carotid** ^**a**^			
Left	1 (2.3%)	2 (4.5%)	0.094
Right	0 (0.0%)	4 (9.1%)	
Both	3 (6.8%)	1 (2.3%)	
**External carotid** ^**a**^			
Left	0 (0.0%)	1 (2.3%)	0.103
Right	0 (0.0%)	0 (0.0%)	
Both	2 (4.5%)	0 (0.0%)	
**Ascending aorta**	7 (15.9%)	5 (11.4%)	0.308
**Aortic arch**	6 (13.6%)	6 (13.6%)	0.735
**Descending aorta**	9 (20.5%)	6 (13.6%)	0.123
**Pulmonary artery**	2 (4.5%)	1 (2.3%)	0.570
**Abdominal aorta**	11 (25%)	5 (11.4%)	0.013
**Axillary artery** ^**a**^			
Left	0 (0.0%)	2 (4.5%)	0.058
Right	0 (0.0%)	3 (6.8%)	
Both	0 (0.0%)	1 (2.3%)	
**Vertebral artery** ^**a**^			
Left	1 (2.3%)	5 (11.4%)	0.182
Right	0 (0.0%)	2 (4.5%)	
Both	2 (4.5%)	2 (4.5%)	
**Renal artery** ^**a**^			
Left	2 (4.5%)	0 (0.0%)	0.269
Right	2 (4.5%)	4 (9.1%)	
Both	4 (9.1%)	4 (9.1%)	
**Iliofemoral artery** ^**a**^			
Left	1 (2.3%)	1 (2.3%)	0.132
Right	2 (4.5%)	0 (0.0%)	
Both	4 (9.1%)	2 (4.5%)	
**Mesenteric**	7 (15.9%)	3 (6.8%)	0.074

^a^Likelihood ratio chi-square test

Table 6 summarizes the types of arterial lesions observed in the samples. The prevalence of dilation and aneurysms was significantly higher in patients with TA and TB than in those with TA without TB (*p* = 0.009 and *p* = 0.027, respectively).

**Table 6:** Types of arterial lesions on CT angiography in patients with Takayasu arteritis with/without tuberculosis

Variables	Tuberculosis		*P* value
	Yes	No	
	*n* (%)	*n* (%)	
**Stenosis**	17 (38.6%)	20 (45.5%)	0.680
**Occlusion**	13 (29.5%)	17 (38.6%)	1.000
**Dilatation**	11 (25.0%)	4 (9.1%)	0.009
**Aneurysms**	7 (15.9%)	2 (4.5%)	0.027

## Discussion

In the case series reported in the literature as well as in our sample, TB was more commonly diagnosed before or concomitantly with the development of TA than during treatment with immunosuppressant medications, as in other autoimmune conditions. Although a direct causal relationship with TB cannot be established, TB may be an infectious trigger for TA onset [10].

Our literature review identified three main studies that compared the characteristics of TA patients with and without a history of TB: two from China and one from the United States.

In a study by Lim et al. of 267 patients with TA, 47 (17.7%) had a history of treatment for TB (34 patients), 10 had been concomitantly diagnosed with TA and TB, and only three developed TB after being diagnosed with TA. No significant intergroup differences were detected in comorbidities, clinical manifestations, or angiographic findings [5].

Another Chinese study evaluated 1105 patients with TA; 109 (9.9%) had a history of TB, 53 (48.6%) had been diagnosed with TB before being diagnosed with TA, and 21.1% had been diagnosed with TB and TA concomitantly. Patients with TB showed significantly greater involvement of the pulmonary artery than those without (31.2% vs. 17.3% without TB) (*p* = 0.001). Chest discomfort, constitutional symptoms, and pulmonary hypertension were the most common symptoms. However, interventional treatment was less frequent in patients with a history of TB. Demographic characteristics, comorbidities, and corticosteroid use rates were similar between the groups. Based on the disease activity assessed using the National Institutes of Health (NIH) score, patients with TB had a higher level of disease activity (61.5%) than those without TB (48.4%) (*p* = 0.01). No significant intergroup differences were noted in acute-phase reagent levels, such as CRP; however, the median ESR was higher in the TB group (*p* = 0.01) [6].

More recently, an observational study conducted by the National Institutes of Health in the United States evaluated 80 patients with TA, among whom ten had a history of TB (eight with LTBI and two with active TB [ganglion + erythema induratum and endometritis]). Patients with TB had a more indolent clinical disease and greater structural aortic damage than those without TB. Despite receiving more aggressive treatments, patients with TB had lower disease recurrence rates [7].

In contrast to Zhang et al., who reported greater pulmonary artery involvement, we found greater abdominal aorta involvement [6]. Our sample’s imaging findings came mostly from CT angiography exams without a specific protocol for analyzing pulmonary artery involvement. Therefore, this involvement may have been underestimated. It was not possible to find a plausible hypothesis for the greater involvement of the abdominal aorta in our study.

A higher degree of dilation and aneurysm prevalence was noted in the TB subgroup in this study. Accordingly, two hypotheses were proposed: some patients classified as having TA may actually have presented with aneurysms due to prior tuberculous aortitis, or this subgroup of patients with more dilations and aneurysms were diagnosed with TA after a longer time with the disease because aneurysms are more common in long-term diseases.

Analysis of active vascular infections with *M. tuberculosis*, also known as vascular TB, revealed the following key characteristics: The most common presentation patterns consisted of saccular aneurysms and pseudoaneurysms, with the main locations being the thoracic (27.6%) and abdominal (20.7%) aortas. Classic aortitis without an aneurysm or stenosing profile (obliterative arteritis) may rarely occur and may be confused with TA [11, 12].

At least three mechanisms have been implicated in the spread of *M. tuberculosis* to large vessels. The first is the extension of a contiguous focus of infection (e.g., dissemination from the pulmonary parenchyma to the thoracic aorta or from the retroperitoneum to the abdominal aorta). The second occurs via the hematogenous route through the implantation of the bacillus in an aorta with pre-existing lesions in the intimal layer (e.g., atherosclerosis). The third mechanism involves septic embolization through the lymphatic vessels of the arterial wall [13, 14].

In untreated arterial TB, the risk of aneurysm rupture is high, and the aneurysm commonly ruptures approximately two months after diagnosis, particularly in cases adjacent to the site of infection [12].

In our two cases of concomitant diagnosis of TA and pulmonary TB, one patient presented with multiple aneurysms, which led to a controversial discussion regarding the difference between TA and TB aortitis [15].

One systematic review of TAK and its association with *M. tuberculosis* demonstrated that out of 13 observational studies, only one failed to detect a link between TAK and TB. This could suggest that infection with Mycobacterium tuberculosis could trigger TAK development. However, data that shows a direct link between them is still lacking [16].

## Conclusion

In our study, patients with TA and a history of TB presented with different symptoms, including a higher prevalence of dilations and aneurysms, which were most commonly found in the abdominal aorta.

## Data Availability

Not applicable.

## Abbreviations

CRP: C-reactive protein
ESR: Erythrocyte sedimentation rate
IGRA: Interferon gamma release assay
LTBI: Latent tuberculosis infection
MT: *Mycobacterium tuberculosis*
TA: Takayasu’s arteritis
TB: Tuberculosis
